# A comprehensive review of HPV vaccination policies in the United States

**DOI:** 10.3389/fpubh.2026.1810845

**Published:** 2026-04-20

**Authors:** Yathreb Bayan Mohamed, Seyed M. Karimi

**Affiliations:** Department of Health Management and Systems Sciences, University of Louisville, Louisville, KY, United States

**Keywords:** consent by minors, HPV policy, HPV vaccination, parental education mandate, pharmacist authority, school-entry requirements, sex education mandate, vaccine purchase program

## Abstract

**Background:**

Human papillomavirus (HPV) is the leading cause of cervical cancer in the United States (U.S.), yet HPV vaccination coverage remains suboptimal and uneven across states. Policy approaches to improve HPV vaccine uptake vary widely across states in terms of health financing, provider authority, school requirements, and consent requirements.

**Methods:**

A comprehensive review of state-level HPV vaccination policies was conducted. Information was identified through peer-reviewed literature (PubMed, Scopus, Google Scholar) and authoritative policy and legislative sources, including the Centers for Disease Control and Prevention, National Alliance of State Pharmacy Associations, National Academy for State Health Policy, and state statutes. Policies were categorized into macro-level health policies (Medicaid expansion, pharmacy and pharmacist authority, and state vaccine purchase programs) and HPV-specific policies (school-entry requirements, classroom sex education mandates, parental education mandates, minor consent laws, and exemption frameworks). Evidence on policy implementation and effectiveness was narratively synthesized.

**Results:**

Substantial heterogeneity in HPV vaccination policy adoption and implementation across U.S. states was documented. Medicaid expansion, pharmacist vaccination authority, and well-enforced school-entry requirements were consistently associated with higher HPV vaccine initiation or completion, while policies with permissive exemptions or weak enforcement demonstrated limited impact. Financing mechanisms such as universal and selective vaccine purchase programs reduced cost barriers but showed inconsistent effects on uptake when implemented in isolation. Evidence suggests that no single policy is sufficient; multi-level, coordinated policy environments yield the greatest improvements in vaccination coverage.

**Conclusion:**

HPV vaccination uptake in the U.S. is shaped by complex and interacting policy mechanisms rather than individual interventions. Integrated policy approaches that combine financing, access expansion, school-based strategies, provider engagement, and enforcement structures are most likely to achieve sustained and equitable improvements in HPV vaccine uptake.

## 1 Introduction

Human papillomavirus (HPV) is considered one of the most common sexually transmitted infections in the United States (U.S.), as approximately 42 million Americans were infected with various types of HPV in 2018, with approximately 13 million new infections each year (1, 2). HPV is associated with multiple cancers, such as cervical, vaginal, oropharyngeal, penile, and anal cancers. Each year, HPV causes approximately 36 thousand cancer cases in all genders (1, 2).

HPV-related diseases can largely be prevented by HPV vaccines, introduced in 2006, and are safe and effective (3, 4). Despite the Centers for Disease Control and Prevention’s (CDC) Advisory Committee on Immunization Practices (ACIP) recommending HPV vaccination, HPV vaccination rates are still low, especially among adolescents in underserved communities in the U.S. (5, 6). Low vaccination uptake in the U.S. may be due to a lack of or ineffective policies, reliance on traditional modes of vaccination delivery through primary care providers, limited healthcare access, and socioeconomic barriers (7).

The landscape of HPV vaccination policies across the U.S. reflects a multifaceted interplay among state legislation, insurance mandates, and public health initiatives, all intended to increase vaccination rates and reduce HPV-related disease incidence (8). This study reviews broad-level health policy interventions that could have affected HPV vaccination in the U.S., as well as HPV-specific policies. Evidence of variations in the implementation of these policies across states is collected and presented. Additionally, the literature on the effectiveness of the policies is reviewed. This review aims to provide a structured and policy-oriented synthesis of HPV vaccination strategies across U.S. states, with a focus on implementation mechanisms, under-vaccinated populations, and access pathways.

It is worth noting that the policy landscape surrounding vaccination is currently evolving. In January 2025, the ACIP proposed revisions to the recommended vaccination schedules, but a federal court order has temporarily paused implementation of these revised recommendations (9). In response, several states have taken independent legislative action to preserve existing vaccination policies. Notably, California Senate Bill 144 (SB 144) was enacted to maintain the HPV vaccination recommendations in place as of January 1, 2025, ensuring continuity of school immunization requirements regardless of federal-level changes (10). More recently, in March 2026, a federal judge issued a stay blocking the 2026 ACIP schedule changes, including revised recommendations for COVID-19, hepatitis B, and other vaccines, following a lawsuit alleging that the Department of Health and Human Services (HHS) bypassed required legal and scientific procedures in appointing new committee members (11). These developments collectively underscore the critical role of state-level policy in safeguarding vaccination coverage and highlight the ongoing tension between federal guidance and state legislative authority, a dynamic that is directly relevant to the policy landscape reviewed in this manuscript.

## 2 Methods

This analysis used a policy review and evidence synthesis approach to examine U.S. state-level human papillomavirus (HPV) vaccination policies and their related implementation strategies. The goal was to identify key policy mechanisms that affect HPV vaccine uptake, including regulatory frameworks, provider authority, laws, financing programs, and system-level interventions. Relevant information was collected through manual searches and multiple resources, including peer-reviewed literature indexed in PubMed, Scopus, and Google Scholar, focusing on HPV vaccination policy and coverage without a year specification to capture all legislative and policy differences across states. In addition, the official public health sources and legislative sources, such as the U.S. CDC, the National Alliance of State Pharmacy Associations (NASPA), state immunization program websites, the National Academy for State Health Policy (NASHP), and state and legislative databases. Moreover, policy briefs and state health department reports on school-entry requirements, universal vaccine purchase (UVP) programs, pharmacy and pharmacist authority, Medicaid expansion, and insurance mandates. The search strategy included key terms such as “HPV vaccination,” “state policy,” “school-entry requirement,” “universal vaccine purchase,” “Medicaid expansion,” “pharmacy authority,” “provider recommendation,” “vaccine uptake,” and “implementation strategies.” Sources were selected that focused only on U.S. policies that describe or evaluate state-level HPV vaccination interventions and their impact on HPV vaccination initiation, completion, or coverage rates.

Additionally, the sources were published in English. Some sources were excluded, including studies conducted outside the U.S., clinical trials unrelated to policy mechanisms, and commentary without policy relevance. No initial publication year restriction was applied; however, empirical studies published between 2006 and 2025 were prioritized.

Policies with potential direct or indirect effects on HPV vaccination were categorized into Medicaid expansion and insurance coverage, pharmacy permission and pharmacist authority, Universal and Selective Vaccine Purchase Programs (UVP/SVP), school policies, and parent-, patient-, and provider-focused interventions. Each policy category was assessed for geographic coverage across states, under-vaccinated population, and implementation characteristics, including exemptions, consent laws, age restrictions, and funding structures.

Extracted data included policy type and scope, states adopting the policy, implementation mechanisms, population coverage, specific age groups, and the policy’s impact on HPV vaccine initiation or completion rates in the U.S. Key data were extracted from each source and organized into thematic tables and narrative summaries. Quantitative data were summarized descriptively when available, such as variation in uptake percentages and rates, while qualitative results were used to contextualize variation in policy effectiveness across states. This analysis depended on publicly available data; therefore, some state-level policy details may be underrepresented, and inconsistencies in reporting may limit comparability.

To improve clarity and usability, extracted policy data were systematically categorized into thematic domains, including Medicaid expansion and vaccine financing policies, school-based policies, and intervention-focused strategies addressing parents, patients, and providers. This structured approach guided the reorganization of the Results section.

## 3 Results

The results are organized into key policy domains to improve clarity and facilitate interpretation. Each subsection presents the main findings, followed by supporting evidence from the included studies. To enable rapid cross-state comparison, a consolidated overview of the main policies with potential effects on HPV vaccination is provided (Table 1). The policies include Medicaid expansion, pharmacist authority, participation in the vaccine purchase program, school-entry requirement, sex education mandate, parental education mandate, consent for minors, and several exemptions. Detailed state-by-state tables for the policy domains are provided in the Supplementary Tables S1–S8.

**Table 1:** Consolidated state-level HPV vaccination policy summary overview.

State/jurisdiction	Medicaid expansion^1^	Pharmacist authority^2^	Vaccine purchase program^3^	School-entry requirement^4^	Sex education mandate^5^	Parental education mandate^6^	Consent by minors^7^	Medical exemption^8^	Religious exemption^9^	Personal exemption^10^
Alabama	Not expanded	Broad	—	Not mandated	Not reported	Not reported	No	Yes	Yes	No
Alaska	Expanded (9/1/2015)	Broad	SVP/UVP hybrid	Not mandated	Not reported	Not reported	No	Yes	Yes	No
Arizona	Expanded (1/1/2014)	Broad	—	Not mandated	Not reported	Not reported	No	Yes	Yes	Yes
Arkansas	Expanded (1/1/2014)	Broad	—	Recommended	Not reported	Not reported	No	Yes	Yes	Yes
California	Expanded (1/1/2014)	Broad	—	Not mandated	Yes	Not reported	Yes	Yes	No	No
Colorado	Expanded (1/1/2014)	Broad	State law/other mechanisms	Not mandated	Not reported	Yes	Yes	Yes	Yes	Yes
Connecticut	Expanded (1/1/2014)	Broad	Select purchase only	Not mandated	Not reported	Not reported	No	Yes	No	No
Delaware	Expanded (1/1/2014)	Broad	—	Recommended	Yes	Not reported	No	Yes	Yes	No
District of Columbia	Expanded	Broad	—	Mandated	Not reported	Not reported	No	Yes	Yes	Yes
Florida	Not expanded	Broad	Select purchase only	Not mandated	No	Not reported	No	Yes	Yes	No
Georgia	Not expanded	Broad	—	Not mandated	Yes	Not reported	No	Yes	Yes	No
Hawaii	Expanded (1/1/2014)	Broad + HPV-Specific	Universal purchase only	Mandated	Yes	Not reported	No	Yes	Yes	No
Idaho	Expanded (1/1/2020)	Broad	Universal purchase only	Not mandated	Not reported	Not reported	Yes	Yes	Yes	Yes
Illinois	Expanded (1/1/2014)	Broad	State law/other mechanisms	Not mandated	Not reported	Yes	Yes	Yes	Yes	No
Indiana	Expanded (2/1/2015)	Broad + HPV-specific	—	Not mandated	No	Yes	No	Yes	Yes	No
Iowa	Expanded (1/1/2014)	Broad + HPV-specific	—	Not mandated	Yes	Yes	No	Yes	Yes	No
Kansas	Not expanded	Broad	—	Not mandated	Yes	Not reported	No	Yes	Yes	No
Kentucky	Expanded (1/1/2014)	Broad	—	Not mandated	Yes	Not reported	No	Yes	Yes	No
Louisiana	Expanded (7/1/2016)	Broad	State law/other mechanisms	Not mandated	Yes	Yes	No	Yes	No	Yes
Maine	Expanded (1/10/2019)	Broad	SVP/UVP hybrid	Not mandated	Yes	Not reported	No	Yes	No	No
Maryland	Expanded (1/1/2014)	Broad	State law/other mechanisms	Not mandated	Yes	Not reported	No	Yes	Yes	No
Massachusetts	Expanded (1/1/2014)	Broad	SVP/UVP hybrid	Not mandated	Not reported	Not reported	No	Yes	Yes	No
Michigan	Expanded (4/1/2014)	Broad	—	Not mandated	No	Yes	No	Yes	Yes	Yes
Minnesota	Expanded (1/1/2014)	Broad	—	Not mandated	Yes	Yes	No	Yes	No	Yes
Mississippi	Not expanded	Broad	State law/other mechanisms	Not mandated	No	Yes	No	Yes	Yes	No
Missouri	Expanded (7/1/2021)	Broad	—	Not mandated	Not reported	Yes	No	Yes	Yes	Yes
Montana	Expanded (1/1/2016)	Broad	—	Not mandated	Yes	Not reported	No	Yes	Yes	No
Nebraska	Expanded (10/1/2020)	Broad	—	Not mandated	Not reported	Not reported	No	Yes	Yes	Yes
Nevada	Expanded (1/1/2014)	Broad	State law/other mechanisms	Not mandated	Yes	Not reported	Yes	Yes	Yes	No
New Hampshire	Expanded (8/15/2014)	Broad	SVP/UVP hybrid	Not mandated	Yes	Not reported	No	Yes	Yes	No
New Jersey	Expanded (1/1/2014)	Broad	—	Not mandated	Yes	Yes	No	Yes	Yes	No
New Mexico	Expanded (1/1/2014)	Broad	SVP/UVP hybrid	Not mandated	Yes	Not reported	No	Yes	Yes	No
New York	Expanded (1/1/2014)	Broad	—	Not mandated	No	Yes	Yes	Yes	No	No
North Carolina	Expanded (12/1/2023)	Broad	—	Not mandated	Yes	Yes	Yes	Yes	Yes	No
North Dakota	Expanded (1/1/2014)	Broad	—	Not mandated	Yes	Not reported	No	Yes	Yes	Yes
Ohio	Expanded (1/1/2014)	Broad	State law/other mechanisms	Not mandated	Yes	Not reported	No	Yes	Yes	Yes
Oklahoma	Expanded (7/1/2021)	Broad	State law/other mechanisms	Not mandated	Not reported	Not reported	No	Yes	Yes	Yes
Oregon	Expanded (1/1/2014)	Broad	SVP/UVP hybrid	Not mandated	Yes	Not reported	Yes	Yes	Yes	Yes
Pennsylvania	Expanded (1/1/2015)	Broad	State law/other mechanisms	Not mandated	No	Not reported	Yes	Yes	Yes	Yes
Rhode Island	Expanded (1/1/2014)	Broad	SVP/UVP hybrid	Mandated	Yes	Not reported	Yes	Yes	Yes	No
South Carolina	Not expanded	Broad	—	Not mandated	Yes	Yes	Yes	Yes	Yes	No
South Dakota	Expanded (7/1/2023)	Broad	Select purchase only	Not mandated	Not reported	Yes	No	Yes	Yes	No
Tennessee	Not expanded	Broad	—	Not mandated	Yes	Not reported	Yes	Yes	Yes	No
Texas	Not expanded	Broad	—	Not mandated	Yes	Yes	No	Yes	Yes	No
Utah	Expanded (1/1/2020)	Broad	—	Not mandated	Yes	Yes	Yes	Yes	Yes	Yes
Vermont	Expanded (1/1/2014)	Broad	SVP/UVP hybrid	Not mandated	Yes	Not reported	No	Yes	Yes	No
Virginia	Expanded (1/1/2019)	Broad	—	Mandated	Not reported	Yes	No	Yes	Yes	Yes
Washington	Expanded (1/1/2014)	Broad	SVP/UVP hybrid	Not mandated	Yes	Yes	Yes	Yes	Yes	Yes
Washington D.C.	Expanded	Broad + HPV-specific	—	Not mandated	Yes	Yes	Yes	Yes	Yes	Yes
West Virginia	Expanded (1/1/2014)	Broad + HPV-specific	State law/other mechanisms	Recommended	Yes	Not reported	No	Yes	No	No
Wisconsin	Not expanded	Broad	Universal purchase only	Not mandated	Not reported	Not reported	No	Yes	Yes	Yes
Wyoming	Not expanded	Broad + HPV-specific	Select purchase only	Recommended	Not reported	Not reported	No	Yes	Yes	No

Sources: ^1^See Supplementary Table S1 for details. ^2^See Supplementary Table S2 for details. ^3^See Supplementary Table S3 for details. ^4^See Supplementary Table S4 for details. ^5^See Supplementary Table S5 for details. ^6^See Supplementary Table S6 for details. ^7^See Supplementary Table S7 for details. ^8^See Supplementary Table S8 for details. ^9^See Supplementary Table S8 for details. ^10^See Supplementary Table S8 for details.

### 3.1 Medicaid expansion

To date, 41 states have expanded their Medicaid programs, which were incentivized under the Affordable Care Act (ACA) (Supplementary Table S1) (12). The expansion reduced the financial burden of healthcare and improved access to preventive care among low-income individuals (13). It also led to an increase in HPV vaccination rates. A couple of studies using data on 13-17-year-old individuals found a 3- to 4-percentage-point increase in HPV vaccine initiation following the Medicaid expansion (14, 15). Another study found a 1.6 percentage-point increase in the HPV vaccine initiation rate among 18-26-year-olds after ACA implementation (16). The increase in HPV vaccine initiation among 18-26-year-old Hispanic individuals was the lowest among all racial and ethnic groups: 0.85 percentage points (16).

#### Section summary

Medicaid expansion under the ACA has been associated with modest but meaningful increases in HPV vaccine initiation, particularly among younger adolescents (a 3–4 percentage-point increase among 13–17-year-olds). However, the persistence of 10 non-expansion states and marked disparities among racial and ethnic minority groups highlight financing policy as an unresolved equity challenge requiring particular attention.

### 3.2 Pharmacy and pharmacist authority in HPV vaccination policy

Having established the foundational role of insurance coverage and financing, the following section examines pharmacy-level policy mechanisms that expand access by broadening the pool of authorized HPV vaccine providers. Pharmacy permission and pharmacist authority for HPV vaccination are two interrelated but distinct policies intended to increase vaccine uptake by increasing the number of vaccine providers and by offering greater convenience for patients whose schedules make it hard to visit traditional providers (17, 18). Pharmacy permission policies refer to state regulations or laws that allow the administration of vaccines, such as HPV, in pharmacies under certain conditions. On the other hand, pharmacist authority policies recognize pharmacists as licensed health care providers who are legally authorized to administer vaccines to eligible groups, extending their role beyond medication dispensing (19, 20). The degree of authority granted to pharmacists to administer vaccines varies widely by state (21). In some states, pharmacists can independently administer all CDC-recommended vaccines to individuals of all ages, while in others, vaccinations are restricted by vaccine type, age limits, and the requirement for a prescription (21, 22).

Policies allowing permission for HPV vaccination in pharmacies generally fall into two categories: first, the broad vaccination policies, which typically authorize pharmacists to administer all CDC-recommended vaccines, including HPV, and second, HPV-specific policies, which impose exclusive restrictions like prescription requirements or age limits only for HPV vaccination (22–24). All 50 states and Washington, D.C, had adopted broad policies as of January 2025 (Supplementary Table S2) (19, 24, 25). A small number of states have HPV-specific rules, often tied to age or prescription requirements, parental consent for minors, or concerns about the vaccine’s association with sexual health (8, 26, 27). Five states (Hawaii, Indiana, Iowa, West Virginia, and Wyoming) and Washington, D.C., specifically list HPV vaccination in law, providing clear pharmacist authority for HPV vaccine administration (Table 2) (8).

**Table 2:** HPV vaccination intervention impact by population group (quality improvement project).

Group	Initiation (pre-post)	Completion (pre-post)	Change in initiation (%)	Change in completion (%)
All patients (9–26)	69.7% → 81.0%	49.5% → 62.0%	11.3%	12.5%
Females	75.0% → 85.6%	59.3% → 70.2%	10.6%	10.9%
Males	58.4% → 71.2%	28.5% → 44.5%	12.8%	16.0%
Adolescents (11–17)	65.3% → 86.5%	35.5% → 55.9%	21.2%	20.4%
Females	63.6% → 84.3%	40.5% → 57.9%	20.7%	17.4%
Males	66.9% → 88.7%	30.6% → 54%	21.8%	23.4%
Young adults (18–26)	75.2% → 82.6%	58.6% → 68.5%	7.4%	9.9%
Females	80.6% → 88.4%	67.6% → 77.1%	7.8%	9.5%
Males	57.9% → 63.9%	30.1% → 41.4%	6.0%	11.3%

Source: McGaffey et al. (92).

One study found a statistically significant increase in HPV vaccine uptake following the implementation of laws granting pharmacists the authority to administer vaccines (28). Another study noted inconsistencies in state laws governing pharmacists’ authority regarding HPV vaccination—for example, allowing pharmacists to vaccinate only older adolescents rather than the CDC-recommended age of 11-12—as a factor hindering the effectiveness of these policies (23). When combined with community-based interventions, including education campaigns, reminders, and standing orders, pharmacy permission and pharmacist authority have been shown to improve HPV vaccine uptake significantly (29–31).

#### Section summary

All 50 states and D.C. now grant pharmacists broad authority to administer HPV vaccines, representing universal baseline access. However, six jurisdictions have enacted HPV-specific statutory authority to clarify this role further. Age-restriction inconsistencies in some states limit pharmacists’ impact on adolescent populations, where the need is greatest. Expanding pharmacist scope—particularly in rural areas—could significantly improve access where primary care physicians are scarce.

### 3.3 State-led vaccine purchase programs

Beyond financing and access mechanisms, states have implemented direct vaccine procurement programs to eliminate cost barriers at the point of delivery. Since the early 1990s, the U.S. Vaccines for Children (VFC) Program has provided federally purchased vaccines to children regardless of their insurance status. In the late 1990s, some states expanded VFC to further increase immunization rates under both private and public insurance plans, thus removing financial barriers to accessing critical preventive healthcare services (15, 32, 33). These state-led funding models show how targeted financing intervention can eliminate cost barriers, expand coverage, and strengthen state leadership in HPV vaccine implementation. Notably, the northeastern U.S. states are national leaders in HPV immunization, with completion rates of 70% or higher among adolescents aged 13–17. One of the main reasons for the high HPV vaccination rates in the regions is their robust vaccine funding mechanisms (8, 34, 35). For example, Maine funds all HPV vaccination at no cost for individuals aged 9–18 through its Universal Childhood Immunization Program (36), while the New Hampshire Immunization Program covers all recommended childhood vaccines, including HPV, for individuals up to 18 years old (37).

Two main state-led funding models are Universal Vaccine Purchase (UVP) and Select Vaccine Purchase (SVP) programs. In a UVP program, a state’s health department purchases all CDC-recommended vaccines, including HPV, and distributes them free of charge regardless of children’s insurance status. States with UVP programs include Alaska, Idaho, Maine, Massachusetts, New Hampshire, New Mexico, Oregon, Rhode Island, Vermont, and Washington (38, 39). In contrast, SVP states such as Connecticut, Florida, South Dakota, and Wyoming purchase and distribute only a subset of the most impactful vaccines (Supplementary Table S3) (8, 38, 39). Some states operate a hybrid model that includes both UVP and SVP programs. For example, in Rhode Island, Vermont, and Washington, all childhood vaccines are universally funded, but only certain adolescent vaccines are selectively funded (Supplementary Table S3). Hybrid models allow more flexibility in managing vaccine financing and optimizing coverage (8, 33).

UVP and SVP programs often operate through public-private partnerships, with funding mechanisms supported by provider participation fees or insurer assessments. Consequently, they extend vaccine access beyond the publicly insured population to include privately insured children and, in some cases, adolescents and adults (8, 33, 40). This integrative approach improves equitable distribution and ensures a sustainable funding stream across states with robust vaccination infrastructure.

The direct impact of the state-led vaccine purchase programs on HPV vaccination coverage remains inconclusive. One study found no significant association between universal purchase programs and overall childhood vaccination uptake rates across ACIP/CDC-recommended vaccines after adjusting for socioeconomic and demographic factors (33). Another study found that the state purchase program for the HPV vaccine was not associated with increased HPV vaccine uptake (41). As a result, financing interventions are considered insufficient; integrative approaches that combine them with communication strategies, provider engagement, and school-entry requirements are more effective at sustaining high HPV vaccine uptake (42).

#### Section summary

State vaccine purchase programs, particularly UVP models, eliminate cost as a direct barrier to HPV vaccination and are associated with the high completion rates seen in northeastern states. However, these financing interventions are not sufficient on their own to drive coverage increases; they achieve their greatest impact when combined with provider engagement, school policies, and community outreach. States without robust purchase programs face compounded access barriers, particularly among uninsured and underinsured populations.

### 3.4 School policies

Having reviewed broad-level financing and access mechanisms, the following sections turn to HPV-specific legislative mandates and their implementation across U.S. states, beginning with the most direct policy tool: school-entry requirements.

#### 3.4.1 School entry requirements

Some states and jurisdictions have mandated HPV vaccination as a school-entry requirement (SER). They are Virginia, the District of Columbia (D.C.), Rhode Island, and Hawaii (43) (Supplementary Table S2). Some other states—namely, West Virginia, Delaware, Wyoming, and Arkansas—have enacted laws that recommend, rather than mandate, HPV vaccination for schoolchildren (8).

Virginia was the first state to adopt a school-entry HPV vaccination requirement in October 2008, initially requiring vaccination for females entering sixth grade. The state later expanded the requirements in July 2021 to include all seventh-grade students, requiring two doses and allowing exemptions for personal beliefs (43–45). The D.C. implemented its mandate in January 2009 for females entering sixth grade and expanded it in December 2014 to include all students, requiring completion of a two- or three-dose series and allowing a personal belief exemption for the HPV vaccine only (44–46). Rhode Island implemented a mandate in 2015 requiring HPV vaccination for all seventh-grade students, consistent with ACIP recommendations and dosing guidelines (8, 43, 44, 47).

Although SERs have the potential to reduce health disparities and increase HPV vaccine uptake (48), evidence on their effects on HPV vaccine uptake remains limited (49). Additionally, SER policies have had different effects on HPV vaccine uptake depending on their enforcement and structure. For example, Washington, D.C., changed its exemption policy by requiring parents to submit a vaccination waiver (opt-out provision) every year rather than just once, leading to a significant increase in vaccination rates: about 11 percentage points among teen girls and 20 percentage points among teen boys (37). Conversely, Virginia’s policy showed little measurable effect on vaccination rates, perhaps due to lenient opt-out phrases and weak enforcement mechanisms (50–54). In contrast, Rhode Island’s more rigorous policy, limited exemptions, and assertive public health outreach have contributed to higher HPV vaccine uptake (43, 47, 55–59). By 2024, states with active SER policies, such as D.C., Rhode Island, and Virginia, reported initiation rates ranging from 86.9 to 91.3% and completion rates ranging from 71.9 to 76.1% among adolescents aged 13–17 years (34).

SERs for other vaccines, like the Tdap booster, have been shown to increase HPV vaccination rates indirectly, suggesting policy spillover effects to other vaccines like the meningococcal conjugate vaccine (MCV) and HPV, as combining vaccine requirements can lead to higher overall coverage in 13-17-year-old female adolescents by 47.3% compared to 42.9% in states without SER (60–64). This indicates how bundling HPV mandates with other adolescent vaccines can strengthen program efficiency and reduce administrative burden among states (35). A recent review confirms these findings, showing a positive association between SER policies and HPV vaccination coverage (42), especially in Washington, D.C, and Rhode Island, where mandates were well enforced and strongly implemented along with other health initiatives (43, 47, 50, 56–58, 65, 66). Moreover, evaluations of adolescent vaccination rates under the SER policies showed a positive spillover effect of SER for Tdap, MenACWY, and Varicella vaccine on HPV vaccination uptake by 4.2 to 2–9 percentage points for the Tdap SER vaccine in 10-13-year-old adolescents (42, 60, 61, 63, 64, 67, 68).

##### Section summary

School-entry requirements represent the most direct HPV-specific mandate available to states. Evidence from four active SER jurisdictions demonstrates that policy design details — particularly exemption stringency, enforcement mechanisms, and integration with other adolescent vaccines — are as consequential as the mandate itself. Rhode Island and D.C.’s rigorous, well-enforced mandates have achieved the highest initiation and completion rates nationally, while Virginia’s lenient enforcement shows that a mandate without a strong implementation infrastructure may fail to achieve its intended impact.

#### 3.4.2 Classroom sex education mandates

While school-entry mandates operate at the institutional level, classroom-level education policies also shape HPV awareness and vaccine acceptance among adolescents and their families. Implementing school sex education mandates in the curriculum has the potential to improve adolescents’ sexual health outcomes, support healthier decision-making, and increase their awareness about sexually transmitted infections and vaccination. These mandates may reduce HPV reluctance among both young individuals and their parents by providing clear, evidence-based information (69, 70).

Classroom sex education mandates related to Human Papillomavirus (HPV) vary widely across U.S. states and carry significant public health implications (Supplementary Table S4) (71). Most states require comprehensive or partial HPV education instructions in school. In contrast, others make it optional or remove it entirely from sex education curriculum, or require only sex education and abstinence instructions, including HIV (72, 73). For clarity, states categorized as having ‘Comprehensive’ sex education mandates are those whose laws explicitly require instruction covering HPV transmission, prevention, and vaccination. States listed as requiring ‘HIV instruction’ may or may not include HCV or broader STI education, where state law was ambiguous (Supplementary Table S5). States are further categorized by whether parental opt-out or opt-in provisions apply, as these exceptions significantly affect practical implementation.

Expanding state-level school-based sex education mandates can be more effective if policies focus on implementation quality, thoughtful program design, and engaging, relevant content (74, 75). However, inconsistency in the extent of classroom sex education mandates limits students’ access to critical educational resources about HPV transmission, prevention, and vaccination, potentially aggravating existing health disparities, particularly among underserved populations with lower vaccination rates (76, 77). Another barrier to implementing sex education mandates is the complexity of integrating comprehensive curricula into diverse school settings, which requires coordination, teacher training, and curriculum adaptation (78). Although conducted outside the U.S., this study highlights implementation challenges that are relevant across diverse educational policy environments.

The relationship between classroom sex education and HPV vaccine uptake is rarely studied, particularly in 13-17-year-old adolescents. The few studies on the subject showed no association (42, 71, 79). However, one study found that educational requirements involving both adolescents aged 9–17 and their parents were associated with 8.8 percentage-point higher HPV vaccine coverage among girls and 8.7 percentage-point higher among boys (41). In addition, a study that included adolescents aged 13–17 years old showed that there is consistency in HPV vaccine initiation associated with classroom sex education mandates (80).

##### Section summary

Classroom sex education mandates exist in various forms across most U.S. states, but their direct impact on HPV vaccine uptake remains limited and inconsistent in the empirical literature. Their most notable effect is observed when education requirements extend to parents as well as students. States seeking to use education policy as a lever for improving HPV coverage should focus on implementation quality, parental engagement, and integration with broader vaccination promotion strategies.

### 3.5 Parents-focused interventions

In addition to institutional school policies, several interventions specifically address parental awareness and decision-making, a critical leverage point given that parental hesitancy remains one of the most frequently cited barriers to adolescent HPV vaccination.

#### 3.5.1 Parental education mandates

Safety concerns have remained one of the most frequently reported reasons for HPV vaccine hesitancy among parents (81). The concerns include fears of neurological disorders, autoimmune conditions, premature ovarian insufficiency, or potential adverse events following vaccination (82, 83). To enhance the HPV awareness and HPV vaccine acceptance among parents, parental education mandates have been included in various states’ legislations (Supplementary Table S6) (8). These mandates require state health departments or school systems to reach out to parents, typically when their children reach middle school, and to ensure that they receive accurate, accessible scientific information about HPV transmission, vaccine safety, and effectiveness, thereby enabling them to make informed vaccination decisions for their children (84).

Although parental education mandates are theoretically expected to improve parental understanding and reduce misinformation and hesitancy, studies evaluating their effectiveness have shown no measurable impact on HPV vaccine uptake (49, 60, 80). These findings suggest that educational mandates alone are insufficient and should be complemented by additional strategies, such as strong provider recommendations, SERs, and community-based engagement, to achieve meaningful increases in vaccination rates.

##### Section summary

Parental education mandates have been adopted in approximately 19 states, reflecting broad recognition that informed parental decision-making is critical to adolescent HPV vaccination. However, empirical evidence consistently shows that these mandates do not, on their own, drive measurable increases in vaccine uptake. They are best understood as a necessary but insufficient component of a multi-strategy approach, most effective when paired with provider-level recommendations and school-entry requirements.

#### 3.5.2 HPV vaccine minor consent policies

Minor consent policies for HPV vaccination vary across the states, with some allowing minors to receive the vaccine without parental consent under specific conditions (Supplementary Table S7). For instance, California permits minors aged 12 and older to consent to HPV vaccination under laws allowing preventive care for sexually transmitted infections (85, 86). Similarly, Illinois and Utah allow minors aged 12 and older to consent, while New York permits HPV vaccination at any age if the healthcare provider determines that the minor is sexually active or may become sexually active (86, 87). Other states, such as Idaho and Washington, follow the “mature minor doctrine,” which allows vaccination if the healthcare provider believes that the minor is capable of giving informed consent (86). In contrast, some states continue to require parental consent for HPV vaccination (86). These policies aim to increase vaccine access for minors while balancing parental involvement and broader public health goals (55, 86). Moreover, improving HPV vaccine uptake requires understanding the broader factors that lead to vaccine refusal and hesitancy among age-appropriate young adults, adolescents, and parents (88).

##### Section summary

At least 15 states allow minors to consent to HPV vaccination without parental approval under specific conditions, expanding access for adolescents facing parental hesitancy. However, most states still require parental consent, and the impact of these policies depends heavily on provider and patient awareness of their existence.

#### 3.5.3 State HPV and general vaccination exemptions laws from school immunization requirements

Beyond policies that expand access to the HPV vaccine, state exemption laws also play an equally important role by determining who may legally opt out of vaccination requirements. This dynamic can significantly affect the real-world impact of school-entry mandates. Most states allow broad exemptions for religious, medical, and/or personal beliefs for vaccination. Immunization exemption laws vary across jurisdictions but generally fall into two categories: specific exemptions for certain vaccines, such as HPV, and broader exemptions, including medical, religious, and personal/philosophical belief exemptions (Supplementary Table S8) (43, 44, 72). Only two jurisdictions, the District of Columbia (D.C.) and Virginia, have specific HPV personal belief exemptions.

Fifty states and D.C. allow medical exemptions with physician approval. In addition, 44 states and D.C. allow religious exemptions, except for six strict states, California, Connecticut, Maine, New York, West Virginia, and (before 2023) Mississippi, that ban all non-medical exemptions (religious or personal). Additionally, 15 states permit personal/ philosophical exemptions, though some restrict their application to childcare settings (43, 44, 72).

Although HPV-specific exemptions are limited to the District of Columbia and Virginia, which permit personal belief exemptions exclusively for HPV vaccination, there has been a trend toward stricter restrictions on non-medical exemptions, especially in states like California, Connecticut, Maine, and New York. Additionally, several states now require parents to complete educational requirements before obtaining non-medical exemptions, an approach aimed at promoting informed decision-making (72).

##### Section summary

Vaccination exemption policies define the practical boundaries of school-entry mandates: states with broad exemption categories or low barriers to obtaining exemptions effectively weaken the force of their mandates. The trend toward tightening non-medical exemption requirements—and in some states, eliminating religious exemptions—reflects a public health response to declining vaccination rates and growing hesitancy. HPV-specific personal belief exemptions, which exist only in D.C. and Virginia, underscore the unique societal sensitivities surrounding this vaccine.

### 3.6 Patient-focused interventions

Having examined the policy and legislative mechanisms that govern access to and exemptions from HPV vaccination, the following sections turn to the behavioral and clinical strategies that addressing patients and providers. It has been shown that adolescents visited by pediatricians have significantly higher HPV vaccine uptake than those seen by family physicians (89, 90). Also, a recent study showed that implementing 10 or more strategies from the 4 Pillars™ Practice Transformation Program (reminder-recall systems, educational outreach, standing orders, provider prompts, audit feedback, and community engagement) was associated with high HPV vaccine uptake among adolescents (91).

A multidimensional quality improvement initiative conducted in an urban, low-income, and minority-serving family medicine clinic in Pittsburgh demonstrated effective results in HPV vaccination initiation and completion. The project, “A ‘Sense’-ational HPV Vaccination Quality Improvement Project”, focused on 9-26-year-old patients to increase their vaccination uptake through the integration of various partnerships, like a multidisciplinary leadership team, community health advocates, health center medical and non-medical staff, including pharmacy residency and family medicine trainees (92). This intervention fostered a welcoming, motivated environment by combining several approaches, including reminder texts, community campaigns, pet therapy, educational materials, standing orders, and other engaging activities as incentives after vaccination.

The effect of that on HPV vaccination initiation rates was observed after 1 year, with an approximately 11.3 percentage-point increase from 69.7 to 81.0%. The HPV vaccine completion rate also increased by 12.5 percentage points: from 49.5 to 62.0% (Table 2) (92). The largest effect of the program was observed among adolescents (11–17-year-olds), with 21.2 and 20.4 percentage-point increases in HPV vaccine initiation and completion, respectively (Table 2) (92). The study highlighted that engaging, tailored, and evidence-based strategies can overcome challenges such as limited access to prevention care, missed opportunities, and vaccination hesitancy (92). The success of this project provides guidance for future HPV policies to include integrated systems and community approaches, and to incorporate a multisensory engagement strategy, particularly for underserved populations, to increase vaccination rates and prevent HPV-related cancers. Research indicates that addressing multiple levels of barriers is critical; providers must develop tailored strategies to overcome parental hesitancy through evidence-based communication and reassurance. To improve U.S. HPV vaccination coverage among racial minorities and other higher-risk groups, combined health policies must integrate community partnerships, provider education, accessible vaccination services, and ongoing system-level support for the initiation and completion of HPV vaccination (93–96).

#### Section summary

Patient-focused, multi-strategy interventions—particularly those combining reminder systems, provider engagement, community partnerships, and accessible vaccination environments—can achieve clinically meaningful increases in both HPV vaccine initiation and completion. The most dramatic gains are seen among adolescents aged 11–17, consistent with the ACIP-recommended vaccination window. These findings highlight the importance of tailored, evidence-based implementation strategies that go beyond policy mandates to address behavioral and access barriers at the individual level.

### 3.7 Provider-focused interventions

Provider-focused interventions address the clinical encounters, as provider recommendations have consistently been shown to be among the strongest determinants of HPV vaccination initiation and completion. However, recommendations are usually not uniformly applied across the full age range of eligible patients (97–104). The greater the providers’ understanding and knowledge of HPV-related cancers (vulva, penis, anus, rectum) and the disease burden, the more likely HPV vaccination will be consistently recommended in accordance with guidelines, which may increase vaccination uptake (102, 103, 105). A study showed that around 56.9% of female 13-17-year-old adolescents were five times more likely to receive the HPV vaccine when healthcare providers recommended it compared to those without a recommendation (97). Exclusively, minorities and other racial groups were strongly associated with HPV vaccination when receiving providers’ recommendations, which shows how important the provider communication and access (97, 106). Studies of other vaccinations, such as hepatitis C and hepatitis B, have shown that provider recommendation is a strong predictor of vaccine acceptance, particularly among older and minority groups, who benefit most from provider engagement (107, 108).

Belief patterns among various specialties differ about the effectiveness of HPV vaccinations. Among various types of providers recommending vaccines, OB/GYNs had the highest rate of vaccine recommendations among females aged 9 to 26, at 29.2%. In comparison, pediatricians recommended females aged 11 to 26 years more frequently (32.4%), and around 18.7% of females aged 18 to 26 years were most frequently recommended by internists (102).

Primary care professionals’ recommendations are considered one of the best practices for increasing HPV vaccination uptake (109). In addition, it has been shown that when patients refuse vaccination, documenting their refusal in electronic health records may increase their future likelihood of accepting the provider’s recommendation, as this provides helpful context to repeat the recommendation (110).

Regarding the effect of pharmacists on access to vaccine as providers, a study that conducted geospatial analysis on the State of Texas found out that the inclusion of pharmacists as vaccine providers could significantly enhance vaccine coverage by 54% in urban areas and 30% in rural census tracts, especially in cities where primary care physician availability is limited, mainly because pharmacists are more widely distributed than primary care physicians (PCPs) (111). These findings suggest that expanding the scope of the pharmacist’s practice could affect access to adolescent vaccinations, including the HPV vaccine, and address key gaps in areas that lack PCPs. In addition, another study found substantial differences in pharmacists’ authority (which varied depending on patient age and state-specific provisions, including requiring a prescription, using a collaborative practice protocol, and granting independent authority) to administer HPV vaccines across U.S. states (30). The study showed that while 80% of states allowed pharmacists to vaccinate adults (ages 19 and older), only 61% permitted vaccinations of adolescents aged 12. This difference in laws affects the pharmacist’s ability to contribute profoundly to cervical cancer prevention strategies (30).

*Section summary*: Provider recommendation is one of the strongest and most consistently identified predictors of HPV vaccine initiation across all age groups, genders, and racial/ethnic backgrounds. Specialty-specific gaps in recommendation patterns, combined with age-restriction inconsistencies in pharmacist authority, represent modifiable barriers to maximizing HPV vaccination coverage. System-level tools—including electronic health record prompts, standing orders, refusal documentation, and expanded pharmacist authority—are evidence-based strategies that can support more consistent and equitable HPV vaccine promotion at the provider level.

## 4 Policy recommendations and conclusion

This review showed that HPV vaccination policy in the U.S. has remained highly heterogeneous, with state-level progress dictated by specific implementation nuances and enforcement mechanisms. While certain policies have proven effective in increasing vaccine initiation, their success is deeply contingent upon the state-specific context. Synthesized evidence indicates that no single policy—whether legislative, clinical, or financial—is sufficient to achieve substantive and equitable population-level coverage. Instead, a robust impact requires a multi-level integration of statutory policies across sectors.

To reduce geographic and social disparities, particularly regarding the disproportionate burden of HPV-related infections and cancers in underserved communities, an integrated policy framework is essential. This framework must harmonize coverage and financing, expand access points, and coordinate provider-patient interventions. Based on this synthesis, an actionable set of recommendations for policymakers, public health practitioners, and researchers includes (1) financial protections and Medicaid expansion, (2) strengthening school-entry requirements, (3) expanding pharmacy-based access, (4) coordinated provider interventions such as electronic health record-based reminder systems, standing orders, and regular audit and feedback programs, (5) vaccine procurement programs, and (6) community-based engagement such as reminder-recall systems and culturally tailored outreach.

## References

[1] Centers for Disease Control and Prevention. About HPV. (2024). Available online at: https://www.cdc.gov/hpv/about/index.html (Accessed February, 12 2026).

[2] Lewis RM Laprise JF Gargano JW Unger ER Querec TD Chesson HW . Estimated prevalence and incidence of disease-associated human papillomavirus types among 15- to 59-year-olds in the United States. Sex Transm Dis. (2021) 48:273–7. doi: 10.1097/OLQ.0000000000001356, 33492097 PMC10037549

[3] De Vincenzo R Conte C Ricci C Scambia G Capelli G. Long-term efficacy and safety of human papillomavirus vaccination. Int J Women's Health. (2014) 6:999–1010. doi: 10.2147/IJWH.S50365, 25587221 PMC4262378

[4] McLemore MR. Gardasil: introducing the new human papillomavirus vaccine. Clin J Oncol Nurs. (2006) 10:559–60. doi: 10.1188/06.CJON.559-560, 17063609

[5] Ventola CL. Immunization in the United States: recommendations, barriers, and measures to improve compliance: part 1: childhood vaccinations. P T. (2016) 41:426–36. 27408519 PMC4927017

[6] Walker TY Elam-Evans LD Yankey D Markowitz LE Williams CL Mbaeyi SA . National, regional, state, and selected local area vaccination coverage among adolescents aged 13-17 years—United States, 2017. MMWR Morb Mortal Wkly Rep. (2018) 67:909–17. doi: 10.15585/mmwr.mm6733a1, 30138305 PMC6107323

[7] Pruitt SL Schootman M. Geographic disparity, area poverty, and human papillomavirus vaccination. Am J Prev Med. (2010) 38:525–33. doi: 10.1016/j.amepre.2010.01.018, 20409501 PMC3259737

[8] Hoss A Meyerson BE Zimet GD. State statutes and regulations related to human papillomavirus vaccination. Hum Vaccin Immunother. (2019) 15:1519–26. doi: 10.1080/21645515.2019.1627817, 31241406 PMC6746494

[9] Wodi APIA Moser CA Cineas S. Advisory Committee on Immunization Practices Recommended Immunization Schedule for Adults Aged 19 Years or Older — United States, 2025, vol. 74 (2025). p. 30–3 doi: 10.15585/mmwr.mm7402a3. PMC11737654 39820474

[10] LARA CICR. BULLETIN 2025–14 (Chaptered Assembly Bill 144 and Coverage of Preventive Care Services). (2025). Available online at: https://www.insurance.ca.gov/0250-insurers/0300-insurers/0200-bulletins/bulletin-notices-commiss-opinion/upload/CDI-Bulletin-2025-14-AB-144-and-preventive-services.pdf (Accessed September 30, 2025).

[11] APHA. Federal Judge Blocks Immunization Schedule Changes, Stays ACIP Member Appointments. APHA (2026).

[12] Kaiser Family Foundation. Status of State Action on the Medicaid Expansion Decision: Kaiser Family Foundation; (2025). Available online at: https://www.kff.org/affordable-care-act/state-indicator/state-activity-around-expanding-medicaid-under-the-affordable-care-act/?currentTimeframe=0&sortModel=%7B%22colId%22:%22Location%22,%22sort%22:%22asc%22%7D

[13] Han X Nguyen BT Drope J Jemal A. Health-related outcomes among the poor: Medicaid expansion vs. non-expansion states. PLoS One. (2015) 10:e0144429. doi: 10.1371/journal.pone.0144429, 26720311 PMC4700996

[14] Hoff BM Livingston MD Thompson EL. The association between state Medicaid expansion and human papillomavirus vaccination. Vaccine. (2020) 38:5963–5. doi: 10.1016/j.vaccine.2020.07.024, 32709431

[15] Churchill BF. Insurance coverage, provider contact, and take-up of the HPV vaccine. Am J Health Econ. (2021) 7:222–47. doi: 10.1086/713037

[16] Gao MZ Awonusi OO Ramkumar SP Myint JA Barnes JM Semprini J . The affordable care act and change in human papillomavirus (HPV) vaccine uptake in the United States. Vaccine. (2025) 50:126842. doi: 10.1016/j.vaccine.2025.126842, 39914253

[17] Goad JA Taitel MS Fensterheim LE Cannon AE. Vaccinations administered during off-clinic hours at a national community pharmacy: implications for increasing patient access and convenience. Ann Fam Med. (2013) 11:429–36. doi: 10.1370/afm.1542, 24019274 PMC3767711

[18] Calo WA Shah PD Gilkey MB Vanderpool RC Barden S Doucette WR . Implementing pharmacy-located HPV vaccination: findings from pilot projects in five U.S. states. Hum Vaccin Immunother. (2019) 15:1831–8. doi: 10.1080/21645515.2019.1602433, 30945968 PMC6746514

[19] Tak CR Marciniak MW Savage A Ozawa S. The essential role of pharmacists facilitating vaccination in older adults: the case of herpes zoster. Hum Vaccin Immunother. (2020) 16:70–5. doi: 10.1080/21645515.2019.1637218, 31369322 PMC7012057

[20] Poudel A Lau ETL Deldot M Campbell C Waite NM Nissen LM. Pharmacist role in vaccination: evidence and challenges. Vaccine. (2019) 37:5939–45. doi: 10.1016/j.vaccine.2019.08.060, 31474520

[21] Bach AT Goad JA. The role of community pharmacy-based vaccination in the USA: current practice and future directions. Integr Pharm Res Pract. (2015) 4:67–77. doi: 10.2147/IPRP.S63822, 29354521 PMC5741029

[22] Mitchell Rae Lynn. States of health: Pharmacist vaccination laws and reaching CDC goals on HPV: Vital Record, Texas A&M Health; (2018). Available online at: https://vitalrecord.tamu.edu/states-health-pharmacist-vaccination-laws-reaching-cdc-goals-hpv/

[23] Dingman DA Schmit CD. Authority of Pharmacists to administer human papillomavirus vaccine: alignment of state Laws with age-level recommendations. Public Health Rep. (2018) 133:55–63. doi: 10.1177/0033354917742117, 29257933 PMC5805097

[24] National Alliance of State Pharmacy Associations. Pharmacist Administred Vaccines: National Alliance of State Pharmacy Associations; (2023). Available online at: https://naspa.us/wp-content/uploads/2021/01/Pharmacist-Immunization-Authority-April-2023.pdf (Accessed February 12, 2026).

[25] 1199 SEIU Benefit Funds. Pharmacist Authority to Administer Vaccines by State. (2019). Available online at: https://www.1199seiubenefits.org/vaccines-by-state/

[26] Essa-Hadad J Gorelik Y Vervoort J Jansen D Edelstein M. Understanding the health system barriers and enablers to childhood MMR and HPV vaccination among disadvantaged, minority or underserved populations in middle- and high-income countries: a systematic review. Eur J Pub Health. (2024) 34:368–74. doi: 10.1093/eurpub/ckad232, 38183166 PMC10990506

[27] Oyedeji O Maples JM Gregory S Chamberlin SM Gatwood JD Wilson AQ . Pharmacists' perceived barriers to human papillomavirus (HPV) vaccination: a systematic literature review. Vaccines (Basel). (2021) 9:1360. doi: 10.3390/vaccines9111360, 34835291 PMC8617618

[28] Trogdon JG Shafer PR Shah PD Calo WA. Are state laws granting pharmacists authority to vaccinate associated with HPV vaccination rates among adolescents? Vaccine. (2016) 34:4514–9. doi: 10.1016/j.vaccine.2016.07.056, 27496275 PMC4996696

[29] Smulian EA Mitchell KR Stokley S. Interventions to increase HPV vaccination coverage: a systematic review. Hum Vaccin Immunother. (2016) 12:1566–88. doi: 10.1080/21645515.2015.1125055, 26838959 PMC4964671

[30] Brewer NT Chung JK Baker HM Rothholz MC Smith JS. Pharmacist authority to provide HPV vaccine: novel partners in cervical cancer prevention. Gynecol Oncol. (2014) 132:S3–8. doi: 10.1016/j.ygyno.2013.12.020, 24361732

[31] Grabenstein JD. Pharmacists as vaccine advocates: roles in community pharmacies, nursing homes, and hospitals. Vaccine. (1998) 16:1705–10. doi: 10.1016/S0264-410X(98)00131-5, 9778745

[32] Freed GL Clark SJ Pathman DE Schectman R Serling J. Impact of North Carolina's universal vaccine purchase program by children's insurance status. Arch Pediatr Adolesc Med. (1999) 153:748–54. doi: 10.1001/archpedi.153.7.748, 10401810

[33] Mulligan K Snider JT Arthur P Frank G Tebeka M Walker A . Examination of universal purchase programs as a driver of vaccine uptake among US states, 1995-2014. Vaccine. (2018) 36:4032–8. doi: 10.1016/j.vaccine.2018.05.103, 29866616 PMC10898222

[34] Zeitouni J Osazuwa-Peters N Dundar Y Zimet G Varvares MA. Two decades of the HPV vaccine: its promise, progress, prospects, projections, and posterity. Lancet Reg Health Am. (2025) 51:101243. doi: 10.1016/j.lana.2025.101243, 41019515 PMC12464959

[35] Pingali C Yankey D Elam-Evans LD Trahan A Markowitz LE DeSisto CL . Vaccination coverage among adolescents aged 13-17 years—national immunization survey-teen, United States, 2024. MMWR Morb Mortal Wkly Rep. (2025) 74:466–72. doi: 10.15585/mmwr.mm7430a1, 40811113 PMC12352626

[36] Maine Legislature. Maine revised statutes annotated. §1066. Universal childhood immunization program (2026). Available online at: https://legislature.maine.gov/statutes/22/title22sec1066-1.html (Accessed 13 February 2026).

[37] Churchill BF. How important is the structure of school vaccine requirement opt-out provisions? Evidence from Washington, DC's HPV vaccine requirement. J Health Econ. (2021) 78:102480. doi: 10.1016/j.jhealeco.2021.102480, 34218042

[38] Fiscus M Cooper R Wilkniss S. Recovering routine immunization rates — state strategies to move beyond COVID-19 National Academy for State Health Policy (NASHP); (2022). Available online at: https://nashp.org/recovering-routine-immunization-rates-state-strategies-to-move-beyond-covid-19/ (Accessed February 13, 2026).

[39] Zhu Y Lin YY Li R He C Lairson DR Deshmukh AA . Reimbursement for HPV vaccine cost in the private sector: a comparison across specialties. Ann Fam Med. (2023) 21:344–6. doi: 10.1370/afm.2990, 37487718 PMC10365861

[40] KidsVax. KV Informational Packet. (2020). Available online at: https://www.kidsvax.org/wp-content/uploads/2020/12/KV-Informational-Packet-2020.pdf

[41] Chen ST Huybrechts KF Bateman BT Hernández-Díaz S. Trends in human papillomavirus vaccination in commercially insured children in the United States. Pediatrics. (2020) 146:e20193557. doi: 10.1542/peds.2019-3557, 32928985

[42] McKeithen MC Gilkey MB Kong WY Oh NL Heisler-MacKinnon J Carlson R . Policy approaches for increasing adolescent HPV vaccination coverage: a systematic review. Pediatrics. (2024) 153:e2023064692. doi: 10.1542/peds.2023-064692, 38623635 PMC11035154

[43] Immunize.org. Exemptions Permitted for State Childcare and School (K–12) Immunization Requirements: Immunize.org; (2024). Available online at: https://www.immunize.org/official-guidance/state-policies/vaccine-requirements/exemptions-child-school-2024/ (Accessed February, 15 2026).

[44] Barraza L Weidenaar K Campos-Outcalt D Yang YT. Human papillomavirus and mandatory immunization Laws: what can we learn from early mandates? Public Health Rep. (2016) 131:728–31. doi: 10.1177/0033354916663184, 28123214 PMC5230814

[45] Stewart A. Childhood vaccine and school entry laws: the case of HPV vaccine. Public Health Rep. (2008) 123:801–3. doi: 10.1177/003335490812300617, 19711662 PMC2556726

[46] Immunize.org. HPV (Human Papillomavirus) Vaccine Requirements for Secondary School: Immunize.org; (2025). Available online at: https://www.immunize.org/official-guidance/state-policies/vaccine-requirements/hpv-secondary-2025/

[47] Washburn T Devi Wold A Raymond P Duggan-Ball S Marceau K Beardsworth A. Current initiatives to protect Rhode Island adolescents through increasing HPV vaccination. Hum Vaccin Immunother. (2016) 12:1633–8. doi: 10.1080/21645515.2016.1161460, 27141954 PMC4964674

[48] Alzahrani MS. Implementing a school-entry mandate for the human papillomavirus vaccine: benefits and challenges. Cureus. (2024) 16:e62519. doi: 10.7759/cureus.62519, 39022520 PMC11253561

[49] Perkins RB Lin M Wallington SF Hanchate AD. Impact of school-entry and education mandates by states on HPV vaccination coverage: analysis of the 2009-2013 National Immunization Survey-Teen. Hum Vaccin Immunother. (2016) 12:1615–22. doi: 10.1080/21645515.2016.1150394, 27152418 PMC4964731

[50] Cuff RD Buchanan T Pelkofski E Korte J Modesitt SP Pierce JY. Rates of human papillomavirus vaccine uptake amongst girls five years after introduction of statewide mandate in Virginia. Am J Obstet Gynecol. (2016) 214:752.e1–6. doi: 10.1016/j.ajog.2016.03.022, 27001221 PMC7740000

[51] Colgrove J Abiola S Mello MM. HPV vaccination mandates — lawmaking amid political and scientific controversy. N Engl J Med. (2010) 363:785–91. doi: 10.1056/NEJMsr1003547, 20818883

[52] Pierre-Victor D Page TF Trepka MJ Stephens DP Li T Madhivanan P. Impact of Virginia's school-entry vaccine mandate on human papillomavirus vaccination among 13-17-year-old females. J Womens Health (Larchmt). (2017) 26:266–75. doi: 10.1089/jwh.2016.5869, 27697003

[53] Wynia MK. Public health, public trust and lobbying. Am J Bioeth. (2007) 7:4–7. doi: 10.1080/15265160701429599, 17558977

[54] Website AcoV. School and Day Care Minimum Immunization Requirements 2025. (2026). Available online at: https://www.vdh.virginia.gov/immunization/requirements/ (Accessed February 12, 2026).

[55] Centers for Disease Control and Prevention. Human papillomavirus (HPV) vaccination: information for health care providers 2021. (2021). Available online at: https://www.cdc.gov/vaccines/hcp/by-disease/hpv.html?CDC_AA_refVal=https%3A%2F%2Fwww.cdc.gov%2Fvaccines%2Fvpd%2Fhpv%2Fhcp%2Frecommendations.html

[56] Curtis CR Dorell C Yankey D Jeyarajah J Chesson H Saraiya M . National human papillomavirus vaccination coverage among adolescents aged 13-17 years-National Immunization Survey--teen, United States, 2011. MMWR Suppl. (2014) 63:61–70. 25208260

[57] Elam-Evans LD Yankey D Jeyarajah J Singleton JA Curtis RC MacNeil J . National, regional, state, and selected local area vaccination coverage among adolescents aged 13-17 years--United States, 2013. MMWR Morb Mortal Wkly Rep. (2014) 63:625–33. 25055186 PMC5779424

[58] Palfrey S. New initiatives to improve HPV vaccination rates. Hum Vaccin Immunother. (2016) 12:1594–8. doi: 10.1080/21645515.2016.1145316, 26930138 PMC4964740

[59] Reagan-Steiner S Yankey D Jeyarajah J Elam-Evans LD Curtis CR MacNeil J . National, regional, state, and selected local area vaccination coverage among adolescents aged 13-17 years—United States, 2015. MMWR Morb Mortal Wkly Rep. (2016) 65:850–8. doi: 10.15585/mmwr.mm6533a4, 27561081

[60] Bugenske E Stokley S Kennedy A Dorell C. Middle school vaccination requirements and adolescent vaccination coverage. Pediatrics. (2012) 129:1056–63. doi: 10.1542/peds.2011-2641, 22566425

[61] Carpenter CS Lawler EC. Direct and spillover effects of middle school vaccination requirements. Am Econ J Econ Policy. (2019) 11:95–125. doi: 10.1257/pol.20170067

[62] Dempsey AF Schaffer SE. Human papillomavirus vaccination rates and state mandates for tetanus-containing vaccines. Prev Med. (2011) 52:268–9. doi: 10.1016/j.ypmed.2010.12.010, 21195727 PMC3062651

[63] Moss JL Reiter PL Rimer BK Brewer NT. Collaborative patient-provider communication and uptake of adolescent vaccines. Soc Sci Med. (2016) 159:100–7. doi: 10.1016/j.socscimed.2016.04.030, 27176467 PMC4881857

[64] Greyson D Vriesema-Magnuson C Bettinger JA. Impact of school vaccination mandates on pediatric vaccination coverage: a systematic review. CMAJ Open. (2019) 7:E524–36. doi: 10.9778/cmajo.20180191, 31431485 PMC6703989

[65] Ko JS Goldbeck CS Baughan EB Klausner JD. Association between human papillomavirus vaccination school-entry requirements and vaccination initiation. JAMA Pediatr. (2020) 174:861–7. doi: 10.1001/jamapediatrics.2020.1852, 32597928 PMC7325070

[66] Falik RB Albrecht SA Cassidy BL. Policy support for expanding the adolescent vaccine school mandate in Pennsylvania to include the human papillomavirus (HPV) vaccine. J Am Assoc Nurse Pract. (2019) 31:263–8. doi: 10.1097/JXX.0000000000000142, 30681651

[67] Potter RC DeVita SF Vranesich PA Boulton ML. Adolescent immunization coverage and implementation of new school requirements in Michigan, 2010. Am J Public Health. (2014) 104:1526–33. doi: 10.2105/AJPH.2014.301910, 24922144 PMC4103225

[68] La EM Garbinsky D Hunter S Poston S Novy P Ghaswalla P. National and state-level composite completion of recommended vaccines among adolescents in the United States, 2015-2018. J Adolesc Health. (2021) 69:762–8. doi: 10.1016/j.jadohealth.2021.07.020, 34518068

[69] Charo RA. Politics, parents, and prophylaxis — mandating HPV vaccination in the United States. N Engl J Med. (2007) 356:1905–8. doi: 10.1056/NEJMp078054, 17494922

[70] Kirby D Laris BA. Effective curriculum-based sex and STD/HIV education programs for adolescents. Child Dev Perspect. (2009) 3:21–9. doi: 10.1111/j.1750-8606.2008.00071.x

[71] Franco M Mazzucca S Padek M Brownson RC. Going beyond the individual: how state-level characteristics relate to HPV vaccine rates in the United States. BMC Public Health. (2019) 19:246. doi: 10.1186/s12889-019-6566-y, 30819149 PMC6393974

[72] National Conference of State Legislatures. State Non-Medical Exemptions From School Immunization Requirements: National Conference of State Legislatures. (2026) Available online at: https://www.ncsl.org/health/state-non-medical-exemptions-from-school-immunization-requirements (Accessed February 15, 2026).

[73] Sex Education Collaborative. (n.d.). Available online at: https://sexeducationcollaborative.org

[74] Denford S Abraham C Campbell R Busse H. A comprehensive review of reviews of school-based interventions to improve sexual-health. Health Psychol Rev. (2017) 11:33–52. doi: 10.1080/17437199.2016.1240625, 27677440

[75] Niland R Flinn C Nearchou F. Assessing the role of school-based sex education in sexual health behaviours: a systematic review. Cogent Psychol. (2024) 11:2309752. doi: 10.1080/23311908.2024.2309752

[76] Silvera SAN Kaplan AM Laforet P. Knowledge of human papillomavirus and cervical Cancer among low-income women in New Jersey. Public Health Rep. (2023) 138:302–8. doi: 10.1177/00333549221081821, 35301894 PMC10031832

[77] Sood RA Carpo BG Leheste JR. Bridging the gap: enhancing HPV (human papillomavirus) education to combat rising Cancer rates. Cureus. (2024) 16:e74023. doi: 10.7759/cureus.74023, 39703290 PMC11658710

[78] Keating S Morgan M Collins B. Relationships and Sexuality Education (RSE) in Primary and Post-Primary Irish Schools. Dublin: The publisher’s name is the National Council for Curriculum and Assessment (NCCA). (2018).

[79] Moghtaderi A Adams S. The role of physician recommendations and public policy in human papillomavirus vaccinations. Appl Health Econ Health Policy. (2016) 14:349–59. doi: 10.1007/s40258-016-0225-6, 26873090

[80] Roberts MC Murphy T Moss JL Wheldon CW Psek W. A qualitative comparative analysis of combined state health policies related to human papillomavirus vaccine uptake in the United States. Am J Public Health. (2018) 108:493–9. doi: 10.2105/AJPH.2017.304263, 29470122 PMC5844399

[81] Sonawane K Zhu Y Montealegre JR Lairson DR Bauer C McGee LU . Parental intent to initiate and complete the human papillomavirus vaccine series in the USA: a nationwide, cross-sectional survey. Lancet Public Health. (2020) 5:e484–92. doi: 10.1016/s2468-2667(20)30139-0, 32707126 PMC7484349

[82] Segal Y Dahan S Calabrò M Kanduc D Shoenfeld Y. HPV and systemic lupus erythematosus: a mosaic of potential crossreactions. Immunol Res. (2017) 65:564–71. doi: 10.1007/s12026-016-8890-y, 28111707

[83] Geier DA Geier MR. Quadrivalent human papillomavirus vaccine and autoimmune adverse events: a case-control assessment of the vaccine adverse event reporting system (VAERS) database. Immunol Res. (2017) 65:46–54. doi: 10.1007/s12026-016-8815-9, 27406735 PMC5406441

[84] Centers for Disease Control and Prevention. Outreach to Parents. (2024). Available online at: https://www.cdc.gov/hpv/php/outreach-to-parents/index.html

[85] Information CL. California Family Code § 6926. (n.d.) Available online at: https://leginfo.legislature.ca.gov/faces/codes_displaySection.xhtml?sectionNum=6926&lawCode=FAM

[86] Teens for Vaccines. Minor Consent Laws for Vaccinations by State (2020). Available online at: https://teensforvaccines.org/minor-consent-laws-by-state/

[87] New York State Department of Health. New York Codes, Rules and Regulations, Title 10, Section 23.4—Minors (n.d.). Available online at: https://regs.health.ny.gov/content/section-234-minors

[88] Fava JP Colleran J Bignasci F Cha R Kilgore PE. Adolescent human papillomavirus vaccination in the United States: opportunities for integrating pharmacies into the immunization neighborhood. Hum Vaccin Immunother. (2017) 13:1844–55. doi: 10.1080/21645515.2017.1325980, 28605256 PMC5557233

[89] Chuang E Cabrera C Mak S Glenn B Hochman M Bastani R. Primary care team- and clinic level factors affecting HPV vaccine uptake. Vaccine. (2017) 35:4540–7. doi: 10.1016/j.vaccine.2017.07.028, 28736202

[90] Irving SA Groom HC Stokley S McNeil MM Gee J Smith N . Human papillomavirus vaccine coverage and prevalence of missed opportunities for vaccination in an integrated healthcare system. Acad Pediatr. (2018) 18:S85–s92. doi: 10.1016/j.acap.2017.09.002, 29502643 PMC6541918

[91] Zimmerman RK Moehling KK Lin CJ Zhang S Raviotta JM Reis EC . Improving adolescent HPV vaccination in a randomized controlled cluster trial using the 4 pillars™ practice transformation program. Vaccine. (2017) 35:109–17. doi: 10.1016/j.vaccine.2016.11.018, 27876200 PMC5836294

[92] McGaffey A Lombardo NP Lamberton N Klatt P Siegel J Middleton DB . A "sense"-ational HPV vaccination quality improvement project in a family medicine residency practice. J Natl Med Assoc. (2019) 111:588–99. doi: 10.1016/j.jnma.2019.06.004, 31285042

[93] Griswold KS Lesko SE Westfall JM. Communities of solution: partnerships for population health. J Am Board Fam Med. (2013) 26:232–8. doi: 10.3122/jabfm.2013.03.130102 23657688

[94] Mansfield LN Vance A Nikpour JA Gonzalez-Guarda RM. A systematic review of human papillomavirus vaccination among US adolescents. Res Nurs Health. (2021) 44:473–89. doi: 10.1002/nur.22135, 33860541 PMC8248517

[95] Thompson EL Best AL Vamos CA Daley EM. "My mom said it wasn't important": a case for catch-up human papillomavirus vaccination among young adult women in the United States. Prev Med. (2017) 105:1–4. doi: 10.1016/j.ypmed.2017.08.016, 28823755

[96] Adjei Boakye E Lew D Muthukrishnan M Tobo BB Rohde RL Varvares MA . Correlates of human papillomavirus (HPV) vaccination initiation and completion among 18-26 year olds in the United States. Hum Vaccin Immunother. (2018) 14:2016–24. doi: 10.1080/21645515.2018.1467203, 29708826 PMC6150015

[97] Ylitalo KR Lee H Mehta NK. Health care provider recommendation, human papillomavirus vaccination, and race/ethnicity in the US National Immunization Survey. Am J Public Health. (2013) 103:164–9. doi: 10.2105/AJPH.2011.300600, 22698055 PMC3518336

[98] Gargano LM Herbert NL Painter JE Sales JM Morfaw C Rask K . Impact of a physician recommendation and parental immunization attitudes on receipt or intention to receive adolescent vaccines. Hum Vaccin Immunother. (2013) 9:2627–33. doi: 10.4161/hv.25823, 23883781 PMC4162064

[99] Brewer NT Gottlieb SL Reiter PL McRee AL Liddon N Markowitz L . Longitudinal predictors of human papillomavirus vaccine initiation among adolescent girls in a high-risk geographic area. Sex Transm Dis. (2011) 38:197–204. doi: 10.1097/olq.0b013e3181f12dbf, 20838362 PMC3025264

[100] Reiter PL Brewer NT Gottlieb SL McRee AL Smith JS. Parents' health beliefs and HPV vaccination of their adolescent daughters. Soc Sci Med. (2009) 69:475–80. doi: 10.1016/j.socscimed.2009.05.024, 19540642

[101] Rosenthal SL Weiss TW Zimet GD Ma L Good MB Vichnin MD. Predictors of HPV vaccine uptake among women aged 19-26: importance of a physician's recommendation. Vaccine. (2011) 29:890–5. doi: 10.1016/j.vaccine.2009.12.063, 20056186

[102] Berkowitz Z Malone M Rodriguez J Saraiya M. Providers' beliefs about the effectiveness of the HPV vaccine in preventing cancer and their recommended age groups for vaccination: findings from a provider survey, 2012. Prev Med. (2015) 81:405–11. doi: 10.1016/j.ypmed.2015.10.007, 26598805 PMC6711477

[103] Daley MF Crane LA Markowitz LE Black SR Beaty BL Barrow J . Human papillomavirus vaccination practices: a survey of US physicians 18 months after licensure. Pediatrics. (2010) 126:425–33. doi: 10.1542/peds.2009-3500, 20679306

[104] Lau M Lin H Flores G. Factors associated with human papillomavirus vaccine-series initiation and healthcare provider recommendation in US adolescent females: 2007 National Survey of children's health. Vaccine. (2012) 30:3112–8. doi: 10.1016/j.vaccine.2012.02.034, 22425179

[105] Centers for Disease Control and Prevention. Basic information about HPV and Cancer (2024). Available online at: https://www.cdc.gov/cancer/hpv/basic-information.html

[106] Bednarczyk RA Birkhead GS Morse DL Doleyres H McNutt LA. Human papillomavirus vaccine uptake and barriers: association with perceived risk, actual risk and race/ethnicity among female students at a New York state university, 2010. Vaccine. (2011) 29:3138–43. doi: 10.1016/j.vaccine.2011.02.045, 21376797

[107] Samoff E Dunn A VanDevanter N Blank S Weisfuse IB. Predictors of acceptance of hepatitis B vaccination in an urban sexually transmitted diseases clinic. Sex Transm Dis. (2004) 31:415–20. doi: 10.1097/01.OLQ.0000130533.53987.78, 15215696

[108] Chi RC Neuzil KM. The association of sociodemographic factors and patient attitudes on influenza vaccination rates in older persons. Am J Med Sci. (2004) 327:113–7. doi: 10.1097/00000441-200403000-00001, 15090748

[109] Oh NL Biddell CB Rhodes BE Brewer NT. Provider communication and HPV vaccine uptake: a meta-analysis and systematic review. Prev Med. (2021) 148:106554. doi: 10.1016/j.ypmed.2021.106554, 33857561

[110] Vielot NA Ballard CAP St Jean DT Page S Hammond K Thompson P . Documenting human papillomavirus vaccine refusal among adolescents in electronic health records: a mixed methods study. Vaccine. (2024) 42:126467. doi: 10.1016/j.vaccine.2024.126467, 39467411

[111] Shah PD Trogdon JG Golden SD Golin CE Marciniak MW Brewer NT. Impact of pharmacists on access to vaccine providers: a geospatial analysis. Milbank Q. (2018) 96:568–92. doi: 10.1111/1468-0009.12342, 30203603 PMC6131320

