# Supplementary material for: A comprehensive review of HPV vaccination policies in the United States
Source: Front Public Health. 2026 Apr 20;14:1810845. doi: 10.3389/fpubh.2026.1810845 (PMC13138340; doi:10.3389/fpubh.2026.1810845)
Supplement: Supplementary file 1 [file Supplementary_file_1.docx]

**A Comprehensive Review of HPV Vaccination Policies in the United States**

Yathreb Bayan Mohamed

Seyed M. Karimi

**Supplemental Appendix**

*Detailed State-by-State Policy Tables (S1–S8)*

The following supplemental tables provide complete state-by-state detail for each policy domain reviewed in the main manuscript. These tables are referenced throughout the Results section by the designations S1–S8. Brief narrative summaries of the key findings from each table are provided within the corresponding subsection of the main manuscript.

**Table S1.** Medicaid Expansion by State and Date

| Date | States Expanded |
| --- | --- |
| 1/1/2014 | Arizona, Arkansas, California, Colorado, Connecticut, Delaware, Hawaii, Illinois, Iowa, Kentucky, Massachusetts, Maryland, Minnesota, Nevada, New Jersey, New York, New Mexico, North Dakota, Ohio, Oregon, Rhode Island, Vermont, Washington, Washington D.C., West Virginia |
| 8/15/2014 | New Hampshire |
| 4/1/2014 | Michigan |
| 1/1/2015 | Pennsylvania |
| 2/1/2015 | Indiana |
| 9/1/2015 | Alaska |
| 1/1/2016 | Montana |
| 7/1/2016 | Louisiana |
| 1/1/2019 | Virginia |
| 1/10/2019 | Maine |
| 1/1/2020 | Idaho |
| 10/1/2020 | Nebraska |
| 1/1/2020 | Utah |
| 7/1/2021 | Oklahoma |
| 7/1/2021 | Missouri |
| 7/1/2023 | South Dakota |
| 12/1/2023 | North Carolina |
| Not Expanded | Alabama, Florida, Georgia, Kansas, Mississippi, South Carolina, Tennessee, Texas, Wisconsin, Wyoming |

*Sources:* Kaiser Family Foundation (2025) (1)

# Table S2. Pharmacy permission /Pharmacists Authority Policies for HPV Vaccination by states

| Policy Category | State |
| --- | --- |
| Macro Vaccination Policy  (General Immunization Authority, HPV allowed under the General law ) | Alabama, Alaska, Arizona, Arkansas, California, Colorado, Connecticut, Delaware, Florida, Georgia, Hawaii, Idaho, Illinois, Indiana, Iowa, Kansas, Kentucky, Louisiana, Maine, Maryland, Massachusetts, Michigan, Minnesota, Mississippi, Missouri, Montana, Nebraska, Nevada, New Hampshire, New Jersey, New Mexico, New York  North Carolina, North Dakota, Ohio, Oklahoma, Oregon, Pennsylvania, Rhode Island, South Carolina, South Dakota, Tennessee, Texas, Utah, Vermont, Virginia, Washington, Washington D.C., Wisconsin, Wyoming |
| HPV- Specific Statutory Authority  (HPV is explicitly listed in law) | Hawaii, Indiana, Iowa, Washington D.C., West Virginia, Wyoming |

# Sources: 1199 SEIU Benefit Funds (2019), Hoss et al. (2019), National Alliance of State Pharmacy Associations (2023), and Roberts et al. (2018) (2-5).

**Table S3**. State HPV Vaccine Purchase Programs

| Policy Type | State | Policy Details |
| --- | --- | --- |
| Select Purchase/ Universal Purchase (Hybrid)( State Law + other mechanisms) | Alaska  Maine  Massachusetts  New Hampshire  New Mexico  Oregon | State programs enable health departments to distribute vaccines to providers for privately insured patients, funded through public-private partnerships. HPV is often included for adolescents. Some cover adults; others only children. |
| State Law/other Mechanisms Only | Colorado  Illinois  Louisiana  Maryland  Mississippi  Nevada  Ohio  Oklahoma  Pennsylvania  West Virginia | Funding through state mechanisms, assessments, or insurer contributions. May not guarantee HPV inclusion statewide. |
| Select Purchase/ Universal Purchase  (Hybrid) | Rhode Island  Vermont  Washington |  |
| Universal Purchase Only | Hawaii  Idaho  Wisconsin | All ACIP-recommended vaccines are purchased and distributed at no cost to providers for pediatric populations. |
| Select Purchase Only | Connecticut  Florida  South Dakota  Wyoming | States purchase only specific vaccines, which may or may not include HPV, often depending on adolescent immunization priorities. |

*Sources:* Hoss et al. (2019), KidsVax (2020), and Zhu et al. (2023)(2, 6, 7)

*Notes:* UVP = Universal Vaccine Purchase. SVP = Select Vaccine Purchase. Programs differ in their inclusion of HPV vaccines, age coverage, and funding mechanisms. Some states operate hybrid UVP/SVP models.

**Table S4.** HPV School-Entry Mandates and Exemptions by State

| State | HPV Vaccine Requirement | Coverage | Date Implemented | Number of Doses Required | Cost to Patient | Exemptions Allowed |
| --- | --- | --- | --- | --- | --- | --- |
| District of Columbia | Yes | Females Entering Grade 6 | 1/2009 | 1, 2, and 3 Doses | Free (VFC / D.C. Program) | Religious, Medical, and Personal Beliefs |
|  | Yes | Males and Females | 12/2014 | 2 and 3 Doses | Free (VFC / D.C. Program) | Religious, Medical, and Personal Beliefs |
| Hawaii | Yes | Students Entering Grade 7 or Higher | 7/2020 | 2 or 3 Doses | Free (VFC Eligible) | Religious and Medical |
|  |  |  | 2015 – Grade 7 | 1 Dose | Free (VFC Eligible) | Religious and Medical |
| Rhode Island | Yes | Males and Females | 2016 – Grade 8  2017 – Grade 9 | 2 Doses  3 Doses (Per ACIP Guidelines) | Free (UVP Program) | Medical Only (Strict Enforcement) |
| Virginia | Yes | Females Entering Grade 6 | 10/2008 | 1, 2, and 3 Doses | Covered By Insurance; VFC For Eligible | Religious, Medical, and Personal Beliefs |
|  | Yes | Males and Females Grade 7 | 7/2021 | 2 Doses (With Dose #1 Prior to Grade 7) | Covered By Insurance; VFC For Eligible | Religious, Medical, and Personal Beliefs |
| Arkansas | Recommended | All Students (Recommended, Not Required) | — | Per ACIP Guidelines | Covered By Insurance; VFC Eligible | Not Mandated |
| Delaware | Recommended | All Students (Recommended) | — | Per ACIP Guidelines | Covered By Insurance; VFC Eligible | Not Mandated |
| West Virginia | Recommended | Recommended For School Children | — | Per ACIP Guidelines | Free (State law Mechanism) | Not Mandated |
| Wyoming | Recommended | Recommended For School Children | — | Per ACIP Guidelines | SVP Program; VFC Eligible | Not Mandated |
| All other states (38) | No | No School-Entry HPV Requirement | — | Per ACIP Guidelines | Insurance / VFC for Eligible | Varies By State |

*Sources:* Barraza et al. (2016), Hoss et al. (2019), Immunize.org (2024), Osazuwa-Peters (2013), Potter et al. (2014), Stewart (2008), Vielot et al. (2020), and Washburn et al. (2016) (2, 8-14).

*Notes:* VFC = Vaccines for Children Program; UVP = Universal Vaccine Purchase; SVP = Select Vaccine Purchase. States with recommended (non-mandated) policies are included for comparative context.

# Table S5. Classroom Sex Education Policies by State

| State | Education Mandated | State Law | Date Implemented | Details of Policy |
| --- | --- | --- | --- | --- |
| California | Yes | § 51933-51934 | 01/1/2016 | Comprehensively Mandated with Parents’ Opt-Out Policy |
| Delaware | Yes | 14, § 851 | 03/01/2020 | Comprehensively Mandated without Parents’ Opt-Out Policy |
| Florida | No | 48-1003.42 | Not Available | Removed |
| Georgia | Yes | §§ 20-2-143 | 07/1/1989 | Partially Mandated with Parents’ Opt-Out |
| Hawaii | Yes | POLICY 103.5 | 16/6/2015 | Comprehensively Mandated with Parents’Opt-Out Policy |
| Indiana | No | § 20-30-5-13 | Not Available | Required HIV Instruction with Parents’Opt-Out Policy |
| Iowa | Yes | § 279.50-§2 56.11  SF 496 | 07/1/1987  26/5/2023 | Comprehensively Mandated with Parents’ Opt-Out Policy  Prohibition Prior 7^th^ Grade |
| Kansas | Yes | § 91-31-32 | 07/1/2005 | Required by Proxy without Parents’ Opt-Out Policy. |
| Kentucky | Yes | 704 KAR 3:305  SB150 156:160 | 2005  2023 | Required by Proxy  Removed Sex Education in Grades 5 and below, with Parents’ Opt-In Policy. |
| Louisiana | Yes | §17:24.4(E) | 8/2014 | Required by proxy with Parents’ Opt-Out Policy |
| Maine | Yes | § 1902-§ 1910 | 2001 | Comprehensively Mandated with Parents’ Opt-Out Policy |
| Maryland | Yes | §7–401 | 07/1/1970 | Comprehensively Mandated with Parents’ Opt-Out Policy |
| Michigan | No | § 380.1507 | 13/1/1977 | Partially Mandated with Parents’ Opt-Out Policy |
| Minnesota | Yes | §§ 121A.23 | 1998 | Comprehensively Mandated with Parents’ Opt-Out Policy |
| Mississipi | No | § 37-13-171 | 1972 | Partially Mandated with Parents’ Opt-In required Policy (abstinence-only) |
| Montana | Yes | §§ 10.53.101/701-709 | Not Available | Required by proxy with Parents’ Opt-Out Policy |
| Nevada | Yes | § 389.036 | 22/5/1979 | Partially Mandated with Parents’ Opt-In Policy |
| New Hampshire | Yes | § 193-E:2-a | 29/6/2007 | Comprehensively Mandated with Parents’ Opt-Out Policy |
| New Mexico | Yes | §§ 22-13-1.1.1  §§ 6.29.6.8  §§ 6.12.2.10 | 2017  24/6/2025 | Comprehensively Mandated with Parents’ Opt-Out Policy |
| New Jersey | Yes | §§ 18A;35-7  §§ 18A;35-8 | 2013 | Comprehensively Mandated with Parents’ Opt-Out Policy |
| New York | No | § 135.3 | Not Available | Partially Mandated with Parents’ Opt-Out Policy |
| North Carolina | Yes | § 115C-81.25  § 115C-81.30 | Not Available | Comprehensively Mandated with Parents’ Opt-In and Opt-Out Required Policy |
| North Dakota | Yes | § 11.1-21-24 | 07/01/2012 | Comprehensively Mandated |
| Ohio | Yes | 3313.60  3313.6011 | 24/10/2024 | Comprehensive Mandated with Parents’ Opt-Out Policy |
| Oregon | Yes | § 336.035  § 336.455 | Not Available | Comprehensively Mandated with Parents’ Opt-Out Policy |
| Pennsylvania | No | § 4.29 | Not Available | Required Abstinence Instruction with Parents’ Opt-out Policy |
| Rhode Island | Yes | §§ 16-1-5  §§ 16-22-17 | Not Available | Comprehensively Mandated with Parents’ Opt-Out Policy |
| South Carolina | Yes | §§ 59-32-10 | 1988 | Comprehensively Mandated with Parents’ Opt-Out Policy |
| Tennessee | Yes | § 49-6-1302  § 49-6-1304 | 2016 | Abstinence only through “Family Life Education” |
| Texas | Yes | §7.102(c)(11) | 30/5/1995 | Required by Proxy with Parents’ Opt-Out Policy |
| Utah | Yes | § 53G-10-402 | 5/7/2025 | Partially Mandated with Parents’ Opt-In Policy |
| Vermont | Yes | §§ 131  §§ 133 | 3/3/1978 | Comprehensively Mandated with Parents’ Opt-Out Policy |
| Washington | Yes | § 28A.300.475 | 11/6/2020 | Comprehensively Mandated with Parents’ Opt-Out Policy |
| Washington D.C. | Yes | § 5-E2304 | 30/12/1994 | Comprehensively Mandated with Parents’ Opt-Out Policy |
| West Virginia | Yes | §§ 18-2-9 | 30/5/1995 | Partially Mandated with Parents’ Opt-Out Policy |

*Sources:* Guttmacher Institute (2025), National Conference of State Legislatures (2020), Perkins et al. (2016), Roberts et al. (2018), and Sex Education Collaborative, n.d. (3, 15-18).

i Data reflect publicly available legislative summaries; district-level implementation may vary.

**Table S6.** HPV Parental Education Mandates

| State | Policy | Year Implemented | Policy Details |
| --- | --- | --- | --- |
| Colorado | Parental Education Mandate |  |  |
| Illinois | Parental Education Mandate  State Law HPV Material (Parents) | 2007 | Requires the Department of Health to Offer 6th-Grade Girls and Parents/Guardians Information on the HPV Vaccine and Its Link to Cervical Cancer. |
| Indiana | Parental Education Mandate  State Law HPV Material (Parents) | 2007 | Requires Parents of 6th-Grade Girls to Receive Information on HPV Vaccine Availability and Its Link to Cervical Cancer. |
| Iowa | Parental Education Mandate | 2007 | Requires a 7th-Grade Education to Include Information on HPV and Vaccine Accessibility |
| Louisiana | Parental Education Mandate  State Law HPV Material (Parents) | 2008  2008 |  |
| Michigan | Parental Education Mandate |  |  |
| Minnesota | State law HPV Materials (Parents) |  |  |
| Mississippi | Parental Education Mandate |  |  |
| Missouri | Parental Education Mandate |  |  |
|  | State law HPV Materials (Parents) |  |  |
| New Jersey | Parental Education Mandate | 2007 | Requires Distribution of Information about HPV to Parents/Guardians |
|  | State law HPV Materials (Parents) |  |  |
| New York | Parental Education Mandate | 2007 | Requires the Department of Health to Offer HPV and Vaccination Info to Parents of Children in Grades 5–12 through Schools |
| North Carolina | Parental Education Mandate  State law HPV Materials (Parents) |  |  |
| South Carolina | Parental Education Mandate |  |  |
| South Dakota | State law HPV Materials (Parents) |  |  |
| Texas | Parental Education Mandate  State law HPV Materials (Parents) | 2007 | Requires Schools to Distribute Accurate, Peer-Reviewed HPV Vaccine Info to Parents/Guardians at the Appropriate Time |
| Utah | Parental Education Mandate |  |  |
| Virginia | Parental Education Mandate |  |  |
|  | State law HPV Materials (Parents) |  |  |
| Washington | Parental Education Mandate | 2007 | Provides Parents of 6th Grade Girls with Information on HPV and Vaccine Accessibility |
|  | State law HPV Materials (Parents) |  |  |
| Washington, D.C. | Parental Education Mandate  State law HPV Materials (Parents) |  |  |

*Sources:* Hoss et al. (2019), Perkins et al. (2016), Roberts et al. (2018), and Sex Education Collaborative, n.d.(2, 3, 15, 17).

*Notes:* Information based on state statutory summaries as of review date.

# Table S7. State Allowing Consent by Minors for HPV or Sexually Transmitted Infections Vaccination

| State | Policy Details |
| --- | --- |
| Alabama | The HPV vaccine is recommended for routine vaccination at age 11-12, and can start at age 9 |
| Alaska | Details unavailable |
| Arizona | Details unavailable |
| Arkansas | Details unavailable |
| California | Minors 12+ in California do not need parental consent for HPV or Hepatitis-B vaccines and can seek care for STDs (Cal. Fam. Code § 6926). |
| Colorado | Colorado SB-21-016 allows minors 12+ to receive STI preventive care, including HPV/Hep-B vaccines, without parental consent |
| Idaho | Idaho allows minors of any age to consent to vaccinations under the ‘mature minor doctrine’ (Idaho Code § 39-4503). |
| Illinois | For HPV and Hepatitis-B vaccines, minors in Illinois 12 years and older in Illinois can consent to vaccinations. |
| Nevada | Minors in Nevada can consent to vaccinations if they meet specific conditions (Nev. Rev. Stat. §129.030), with AB 197 proposing additional amendments |
| New York | Minors in New York can consent to HPV vaccines if sexually active or contemplating it, as per 10 NYCRR §23.4 |
| North Carolina | For non-COVID-19 vaccines, minors of any age in North Carolina can consent to vaccinations (N.C. Gen. Stat. § 90‐21.5). |
| Oregon | Minors 15+ in Oregon can consent to vaccinations (Or. Rev. Stat. §109.640), and minors of any age can receive STI treatment without parental consent (Or. Rev. Stat. §109.610). |
| Pennsylvania | Minors of any age in Pennsylvania who are married, high school graduates, or have been pregnant can consent to all vaccinations (Act of Feb. 13, 1970, P.L. 19, No. 10 Cl. 35) |
| Rhode Island | Minors in Rhode Island aged 16 years and above can consent to vaccinations. |
| South Carolina | Minors in South Carolina, ages 16 years and above, can consent to all vaccinations (SC Code § 63-5-340) |
| Tennessee | Minors 14+ in Tennessee can consent to all vaccinations if deemed a ‘mature minor’ by a healthcare provider (TN Mature Minor Doctrine) |
| Utah | Minors in Utah cannot consent to vaccinations, except for pregnant minors, minor parents, those claiming abandonment for HPV, and unaccompanied homeless minors 15+ (Utah Code §78B-3-406(6)(k)). |
| Washington | Minors of any age in Washington can consent to immunizations under the Mature Minor Doctrine if deemed mature enough by a healthcare provider. |
| Washington D.C | Minors 11+ in Washington, D.C., can consent to all ACIP-recommended vaccines if they meet the informed consent standard (D.C. Law 23-193) |

*Sources*: Chen et al. (2012), Coleman and Rosoff (2013), Hoss et al. (2019), and Teens for Vaccines (2020)(2, 19-21)

# Table S8. State HPV and General Vaccination Exemptions Laws

|  | Type of Exemption | | |  |
| --- | --- | --- | --- | --- |
| State | Medical | Religious | Personal | Comment |
| Alabama | Yes | Yes | No |  |
| Alaska | Yes | Yes | No |  |
| Arizona | Yes | Yes | Yes | Religious: Childcare Only ; Personal Belief : K-12 Only  Parent/guardian must complete the online course for a non-medical exemption. |
| Arkansas | Yes | Yes | Yes | Parent/guardian must complete the online course for a non-medical exemption. |
| California | Yes | No | No | California removed its personal and religious exemption option in 2015. |
| Colorado | Yes | Yes | Yes | Parent/guardian must complete the online course for a non-medical exemption. |
| Connecticut | Yes | No | No | CT ended religious exemptions in 2021; those granted before Apr 28 are valid through 12th grade. |
| Delaware | Yes | Yes | No |  |
| District of Columbia | Yes | Yes | Yes | Personal belief: allowed for HPV only |
| Florida | Yes | Yes | No |  |
| Georgia | Yes | Yes | No |  |
| Hawaii | Yes | Yes | No | Religious exemption requires a provider-signed certificate from the parent/guardian. |
| Idaho | Yes | Yes | Yes |  |
| Illinois | Yes | Yes | No |  |
| Indiana | Yes | Yes | No |  |
| Iowa | Yes | Yes | No |  |
| Kansas | Yes | Yes | No |  |
| Kentucky | Yes | Yes | No |  |
| Louisiana | Yes | No | Yes |  |
| Maine | Yes | No | No | ME ended religious/personal exemptions in 2019; those held before Sep 1, 2021, remain valid if certain requirements are met. |
| Maryland | Yes | Yes | No |  |
| Massachusetts | Yes | Yes | No |  |
| Michigan | Yes | Yes | Yes |  |
| Minnesota | Yes | No | Yes | Childcare only: centers have the option not to enroll a child with a non-medical exemption  Personal exemption requires a notarized statement of conscientiously held beliefs. |
| Mississippi | Yes | Yes | No | MS allowed religious exemptions per the April 2023 court order; parent/guardian must watch the educational video at the county health department. |
| Missouri | Yes | Yes | Yes | Personal belief: childcare only |
| Montana | Yes | Yes | No |  |
| Nebraska | Yes | Yes | Yes | Personal belief: childcare only |
| Nevada | Yes | Yes | No |  |
| New Hampshire | Yes | Yes | No |  |
| New Jersey | Yes | Yes | No |  |
| New Mexico | Yes | Yes | No |  |
| New York | Yes | No | No | New York removed its religious exemption option in 2019. |
| North Carolina | Yes | Yes | No |  |
| North Dakota | Yes | Yes | Yes |  |
| Ohio | Yes | Yes | Yes |  |
| Oklahoma | Yes | Yes | Yes |  |
| Oregon | Yes | Yes | Yes | Non-medical exemption requires provider-signed request or certificate after completing educational module. |
| Pennsylvania | Yes | Yes | Yes |  |
| Rhode Island | Yes | Yes | No |  |
| South Carolina | Yes | Yes | No |  |
| South Dakota | Yes | Yes | No |  |
| Tennessee | Yes | Yes | No |  |
| Texas | Yes | Yes | No |  |
| Utah | Yes | Yes | Yes |  |
| Vermont | Yes | Yes | No | Parent/guardian must review evidence-based educational material to receive a religious exemption. |
| Virginia | Yes | Yes | Yes | Personal belief: allowed for HPV only |
| Washington | Yes | Yes | Yes | Personal belief: not allowed for MMR |
| West Virginia | Yes | No | No |  |
| Wisconsin | Yes | Yes | Yes |  |
| Wyoming | Yes | Yes | No |  |

*Sources:* Barraza et al. (2016), Immunize.org (2024), and National Conference of State Legislatures (2026) (8, 9, 22).

**References:**

1. Kaiser Family Foundation. Status of State Action on the Medicaid Expansion Decision: Kaiser Family Foundation; 2025 [Available from: <https://www.kff.org/affordable-care-act/state-indicator/state-activity-around-expanding-medicaid-under-the-affordable-care-act/?currentTimeframe=0&sortModel=%7B%22colId%22:%22Location%22,%22sort%22:%22asc%22%7D>.

2. Hoss A, Meyerson BE, Zimet GD. State statutes and regulations related to human papillomavirus vaccination. Hum Vaccin Immunother. 2019;15(7-8):1519-26.

3. Roberts MC, Murphy T, Moss JL, Wheldon CW, Psek W. A Qualitative Comparative Analysis of Combined State Health Policies Related to Human Papillomavirus Vaccine Uptake in the United States. Am J Public Health. 2018;108(4):493-9.

4. National Alliance of State Pharmacy Associations. Pharmacist Administred Vaccines: National Alliance of State Pharmacy Associations; 2023 [Available from: <https://naspa.us/wp-content/uploads/2021/01/Pharmacist-Immunization-Authority-April-2023.pdf>.

5. 1199 SEIU Benefit Funds. Pharmacist Authority to Administer Vaccines by State 2019 [Available from: <https://www.1199seiubenefits.org/vaccines-by-state/>.

6. KidsVax. KV informational packet 2020 [Available from: <https://www.kidsvax.org/wp-content/uploads/2020/12/KV-Informational-Packet-2020.pdf>.

7. Zhu Y, Lin YY, Li R, He C, Lairson DR, Deshmukh AA, Sonawane K. Reimbursement for HPV Vaccine Cost in the Private Sector: A Comparison Across Specialties. Ann Fam Med. 2023;21(4):344-6.

8. Barraza L, Weidenaar K, Campos-Outcalt D, Yang YT. Human Papillomavirus and Mandatory Immunization Laws: What Can We Learn From Early Mandates? Public Health Rep. 2016;131(5):728-31.

9. Immunize.org. Exemptions Permitted for State Childcare and School (K–12) Immunization Requirements: Immunize.org; 2024 [Available from: <https://www.immunize.org/official-guidance/state-policies/vaccine-requirements/exemptions-child-school-2024/>.

10. Potter RC, DeVita SF, Vranesich PA, Boulton ML. Adolescent immunization coverage and implementation of new school requirements in Michigan, 2010. Am J Public Health. 2014;104(8):1526-33.

11. Osazuwa-Peters N. Human papillomavirus (HPV), HPV-associated oropharyngeal cancer, and HPV vaccine in the United States--do we need a broader vaccine policy? Vaccine. 2013;31(47):5500-5.

12. Stewart A. Childhood vaccine and school entry laws: the case of HPV vaccine. Public Health Rep. 2008;123(6):801-3.

13. Vielot NA, Butler AM, Trogdon JG, Ramadas R, Smith JS, Eyler A. Association of State Legislation of Human Papillomavirus Vaccination with Vaccine Uptake Among Adolescents in the United States. J Community Health. 2020;45(2):278-87.

14. Washburn T, Devi Wold A, Raymond P, Duggan-Ball S, Marceau K, Beardsworth A. Current initiatives to protect Rhode Island adolescents through increasing HPV vaccination. Hum Vaccin Immunother. 2016;12(6):1633-8.

15. Sex Education Collaborative. n.d. [Available from: <https://sexeducationcollaborative.org>.

16. National Conference of State Legislatures. State Policies on Sex Education in Schools: National Conference of State Legislatures; 2020 [Available from: <https://www.ncsl.org/health/state-policies-on-sex-education-in-schools>.

17. Perkins RB, Lin M, Wallington SF, Hanchate AD. Impact of school-entry and education mandates by states on HPV vaccination coverage: Analysis of the 2009-2013 National Immunization Survey-Teen. Hum Vaccin Immunother. 2016;12(6):1615-22.

18. Guttmacher Institute. Sex Education and HIV Education: Guttmacher Institute; 2025 [Available from: <https://www.guttmacher.org/state-policy/explore/sex-and-hiv-education>.

19. Chen DT, Shepherd L, Becker DM. The HPV vaccine and parental consent. Virtual Mentor. 2012;14(1):5-12.

20. Coleman DL, Rosoff PM. The legal authority of mature minors to consent to general medical treatment. Pediatrics. 2013;131(4):786-93.

21. Teens for Vaccines. Minor Consent Laws for Vaccinations by State 2020 [Available from: <https://teensforvaccines.org/minor-consent-laws-by-state/>.

22. National Conference of State Legislatures. State Non-Medical Exemptions From School Immunization Requirements: National Conference of State Legislatures. ; 2026 [Available from: <https://www.ncsl.org/health/state-non-medical-exemptions-from-school-immunization-requirements>.
